# Dissection of Factors Affecting the Variability of the Peptide Bond Geometry and Planarity

**DOI:** 10.1155/2017/2617629

**Published:** 2017-10-15

**Authors:** Nicole Balasco, Luciana Esposito, Amarinder Singh Thind, Mario Rosario Guarracino, Luigi Vitagliano

**Affiliations:** ^1^Istituto di Biostrutture e Bioimmagini, CNR, Napoli, Italy; ^2^Istituto di Calcolo e Reti ad Alte Prestazioni, CNR, Napoli, Italy

## Abstract

Proteins frequently assume complex three-dimensional structures characterized by marginal thermodynamic stabilities. In this scenario, deciphering the folding code of these molecular giants with clay feet is a cumbersome task. Studies performed in last years have shown that the interplay between backbone geometry and local conformation has an important impact on protein structures. Although the variability of several geometrical parameters of protein backbone has been established, the role of the structural context in determining these effects has been hitherto limited to the valence bond angle *τ* (NC^*α*^C). We here investigated the impact of different factors on the observed variability of backbone geometry and peptide bond planarity. These analyses corroborate the notion that the local conformation expressed in terms of (*ϕ*, *ψ*) dihedrals plays a predominant role in dictating the variability of these parameters. The impact of secondary structure is limited to bond angles which involve atoms that are usually engaged in H-bonds and, therefore, more susceptible to the structural context. Present data also show that the nature of the side chain has a significant impact on angles such as NC^*α*^C^*β*^ and C^*β*^C^*α*^C. In conclusion, our analyses strongly support the use of variability of protein backbone geometry in structure refinement, validation, and prediction.

## 1. Introduction

Proteins are large macromolecules that play a primary role in all biological processes. It is commonly assumed that their functions are strictly related to the three-dimensional structural organization of the constituent atoms. With the exception of rather few highly stable proteins, protein structured states, even when nontransient, are marginally stable. The simultaneous complexity and fragility of these structures make proteins a sort of giants with clay feet. These considerations clearly explain the difficulties encountered in the last decades to decipher the protein folding code [1–3]. Indeed, an appropriate description of protein structures should properly account for a huge number of different energetic factors, some of which have been identified only recently. Indeed, protein folding is the delicate balance of several distinct factors which include well known (H-bonds, electrostatic, and hydrophobic) and more recently discovered (*n* → *π*^*∗*^ interactions, intraresidue H-bonds) determinants [4, 5]. Studies carried out in the last two decades have shown that the interplay between local conformation and protein backbone geometry has important structural consequences [6–22], even in highly restrained contexts [6]. In this framework, it has been shown that local geometry has a crucial impact on allowing/disallowing specific conformations [23, 24]. Moreover, we have shown that the optimization of backbone geometry and local conformation provides an important contribution to the protein stability [7, 13]. Indeed, quantum mechanics calculations have shown that swapping geometrical parameters between different accessible conformations has an energetic cost of 1-2 kcal/mol* per* residue [7]. It has also been highlighted that the optimization of protein geometry may be important for improving protein structure prediction [25, 26].

The variability of protein backbone geometry involves different parameters. These include bond distances, bond angles, and dihedral angles. Initial investigations have highlighted the conformational-dependent variability of the *τ* (NC^*α*^C) angle [9, 14]. Subsequent studies have extended this concept to the other backbone valence bond angles, to the peptide bond planarity, and, more recently, to bond distances [7, 8].

Many efforts have been made to unravel the factors that, besides conformation, may have an impact on *τ* angle. These investigations led to the conclusions that other factors such as secondary structure and residue type may affect the value of this angle, though at lower extent compared to conformation [9, 27–29]. Using statistical analyses of a recent ensemble of structures retrieved from the Protein Data Bank (PDB), we here extended these analyses to the other protein backbone parameters. Moreover, we also evaluated the dependence of peptide bond distortion (in terms of variations of the omega angle from planarity) and carbon carbonyl pyramidalization from the local structural context.

## 2. Methods

Statistical surveys of peptide bond geometrical parameters (bond and dihedral angles) were performed on ensembles of protein structures reported in the PDB (release of March 2016). These structures were selected using the PISCES culling server (http://dunbrack.fccc.edu/PISCES.php) applying specific criteria: resolution better than 1.6 Å for bond angles (Data 1.6) or 1.2 Å for dihedral angles (Data 1.2), sequence identity ≤ 25%, and *R*-factor ≤ 0.20 [30]. Additional selections of the structures of these datasets were carried out at residue level. In particular, in order to reduce local inaccuracies, we excluded the residues for which the ratio between the average backbone *B*-factor (atomic displacement parameter) of the residue and the same parameter calculated considering the entire chain was higher than 1.3. Data 1.6 and Data 1.2 datasets contain 3291 and 799 nonredundant protein chains, respectively.

**Figure 1:**
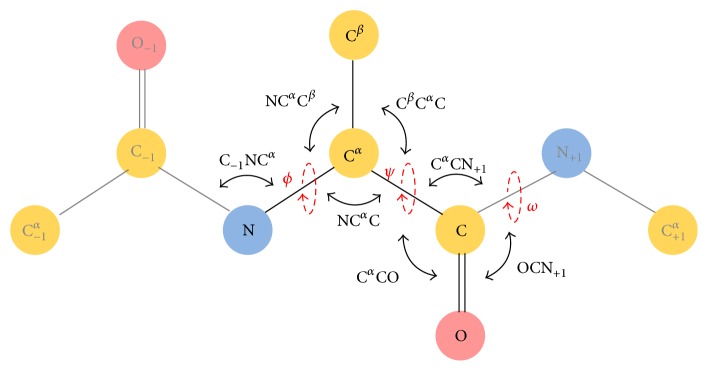
Schematic representation that shows the standard backbone torsion angles *φ*, *ψ*, and *ω* and the bond angles NC^*α*^C, C^*β*^C^*α*^C, NC^*α*^C^*β*^, C^*α*^CO, C^*α*^CN^+1^, OCN^+1^, and C^−1^NC^*α*^. The dihedral angle *ω*3, which is used for the definition of the carbon carbonyl pyramidalization (see Methods), is defined by the atoms OCN^+1^C^*α*+1^.

The analyses dealt with all six bond angles involving non-H atoms of the protein backbone (C^*β*^C^*α*^C, NC^*α*^C^*β*^, C^*α*^CO, C^*α*^CN^+1^, OCN^+1^, and C^−1^NC^*α*^) and two parameters that describe the peptide bond distortions (Δ*ω* and *θ*_C_) (Figure 1). In particular, Δ*ω* defined as (*ω*  −180°) mod 360° represents the peptide bond deviations from planarity [12], whereas *θ*_C_ measured as (*ω* − *ω*_3_  +180°) mod 360° (with *ω*_3_ being the dihedral angle defined by the atoms OCN^+1^C^*α*+1^) describes the displacement of the carbonyl carbon atom from the plane defined by its three bonded atoms (C^*α*^, O, and N^+1^) known as carbonyl carbon pyramidalization [10]. Some of the analyses were performed by computing average values of the geometrical parameters in specific (*ϕ*, *ψ*) boxes of the Ramachandran plot. In order to avoid the mixing of heterogeneous residues in terms of conformation, we minimized the size of these areas as much as possible while ensuring, at the same time, a significant number of observations.

The DSSP program [31] was used for the assignment of secondary structure elements as *α*-helix (H), 3(10)-helix (G), and *β*-sheet (E). Residues with a different notation (all but H, G, and E) were classified as coil (C). The statistical significance of the differences between the average values of pairs of angle distributions was evaluated assuming the so-called null hypothesis (no difference between the mean values) in a two-sample *t*-test analysis.

## 3. Results and Discussion

We initially evaluated the variability of both valence bond geometry and peptide bond planarity in the Ramachandran space using a recent database of protein structure (see Methods for details). In line with previous analyses [7, 14], we considered the bond angles formed by nonhydrogen atoms of the protein backbone (NC^*α*^C, C^*β*^C^*α*^C, NC^*α*^C^*β*^, C^*α*^CO, C^*α*^CN^+1^, OCN^+1^, and C^−1^NC^*α*^) and Δ*ω* and *θ*_C_ as indicators of the peptide bond distortions from planarity (Figure 1). Initial analyses were conducted by considering all non-Gly/non-Pro residues in all types of structures in recent protein structure ensembles (Data 1.6 and Data 1.2 for bond and dihedral angles, resp.; see Methods for details), as Pro and Gly frequently display peculiar structural properties at geometry level [9, 29]. As shown in Figure S1 (in Supplementary Material available online at https://doi.org/10.1155/2017/2617629), all of the considered parameters display significant variability in the Ramachandran space. The comparison of these figures with those previously reported in the literature [7, 11–14] indicates a very close agreement. This observation clearly indicates that the increased size of the current databases does not have an impact on literature trends. Nevertheless, its larger content of structural information allows a more appropriate dissection of the possible factors influencing these variabilities.

As detailed in the following sections, for both backbone geometry and peptide bond planarity distortions, we evaluated the impact of the local (*φ*, *ψ*) conformation and of the structural context (occurrence of a specific secondary structure motif). For the backbone geometry, we also monitored the impact of the residue type on the observed variability.

### 3.1. Backbone Variability: Conformation versus Structural Context

**Figure 2:**
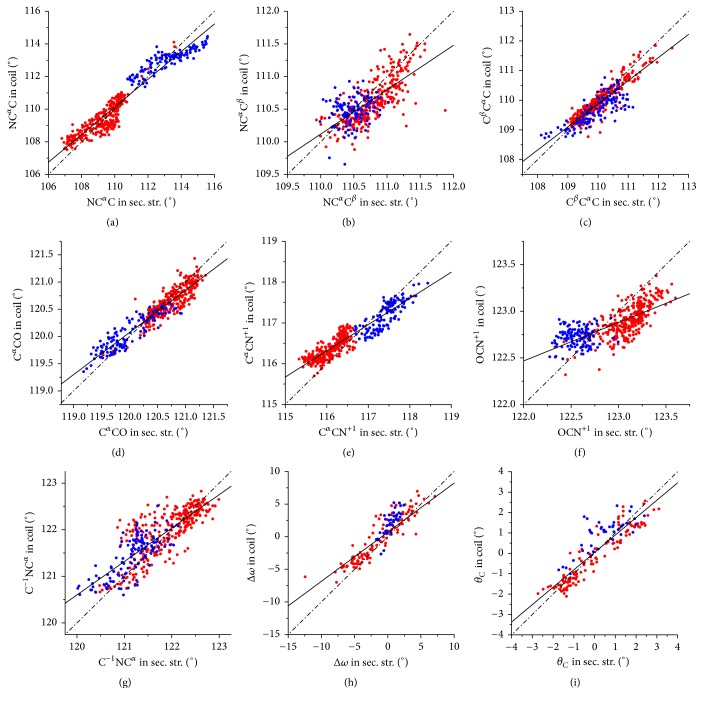
Correlation between the average values of each geometrical parameter computed in the same (*ϕ*, *ψ*) boxes of residues belonging to secondary structure elements (*β*-structure in red and *α*-helices in blue) with those of residues of coil structures: NC^*α*^C (a), NC^*α*^C^*β*^ (b), C^*β*^C^*α*^C (c), C^*α*^CO (d), C^*α*^CN^+1^ (e), OCN^+1^ (f), C^−1^NC^*α*^ (g), Δ*ω* (h), and *θ*_C_ (i). Continuous and dashed-dotted lines are used for the regression lines and diagonals (*y* = *x*), respectively.

These analyses were conducted by dissecting the Ramachandran space in (*ϕ*, *ψ*) boxes and considering collectively all eighteen non-Pro/non-Gly residues. In those boxes that were sufficiently populated (at least 50 residues* per* box), we separately evaluated the average values for each parameter for either residues belonging to secondary structure elements or residues embedded in nonregular regions. The correlation between the values computed in the same (*ϕ*, *ψ*) box is reported for the different parameters (NC^*α*^C, NC^*α*^C^*β*^, C^*β*^C^*α*^C, C^*α*^CO, C^*α*^CN^+1^, OCN^+1^, C^−1^NC^*α*^, Δ*ω*, and *θ*_C_) in Figures 2(a)–2(i). The values of the correlation coefficients and regression line parameters are reported in Table 1. An overview of these figures and of the correlation coefficients suggests that all these parameters tend to adopt similar values in different structural contexts (secondary structure or coil). More specifically, as for NC^*α*^C [29], the valence bond angles C^*α*^CO, C^*α*^CN^+1^, C^*β*^C^*α*^C, and C^−1^NC^*α*^ exhibit very good agreements (correlation coefficient > 0.83) between the two ensembles. Indeed, the continuous fitting lines reported in Figures 2(a)–2(i) suggest that the variability of these parameters follows the same trends in the two distinct contexts. Moreover, the dashed-dotted diagonal line (*y* = *x*) indicates that also the absolute values of these bond angles are rather similar.

The correlation observed for NC^*α*^C^*β*^ and OCN^+1^, though highly significant, is less optimal. It is worth noting, however, that these latter angles display a limited overall viability (~3° for NC^*α*^C^*β*^ and ~1.7° for OCN^+1^; see Figure S1). Moreover, they also involve nitrogen and/or oxygen atoms whose position may be influenced by the local structural context being H-bond formers.

The analysis of the parameters that measure the deviations from planarity of the peptide bond indicates that for both Δ*ω* and *θ*_C_ the structural environment plays a marginal role.

These observations clearly demonstrate that the local conformation is the predominant factor in determining the values of these geometrical parameters as residues in boxes with the same (*ϕ*, *ψ*) values but embodied in different structural contexts display rather similar values. This indicates that the general variability of peptide bond planarity is an intrinsic feature of the local conformation of the polypeptide chain.

Regression lines and correlation coefficients were also calculated separately for *α*-helix and *β*-sheet structures. As reported in Table S1, *β*-structures present very high correlation coefficients for all parameters. Highly significant correlation coefficients are generally exhibited also by *α*-helical residues. The two exceptions are the angles NC^*α*^C^*β*^ and OCN^+1^ whose coefficients present either a limited (NC^*α*^C^*β*^) or no (OCN^+1^) statistical significance. This finding is not surprising taking into account the very limited variability of these two parameters in the helical regions.

To assess the role (if any) of secondary structure and to dissect the relative impact of structure and conformation, we performed additional analyses by comparing the mean values of each geometrical parameter of non-Gly/non-Pro residues in specific boxes of the Ramachandran space. The impact of the secondary structure was evaluated by comparing the average values of these parameters for residues either in coil region or in secondary structure elements (*α*-helix and *β*-structure). To maximize the significance of these analyses, we selected the most populated regions of the plot. In particular, we considered the boxes 3°  × 3° centered at (*ϕ*, *ψ*) = (−63°, −43°) and 15°  × 15° centered at (*ϕ*, *ψ*) = (−120°, 130°) corresponding to helical and extended states, respectively. It is worth mentioning that the standard deviations observed for these parameters in each box (Tables 2 and 3) are significantly lower than those associated in the Engh and Huber parameters [32], which are commonly used in protein refinement protocols (Table S2). This discrepancy is not surprising since the Engh and Huber analysis did not consider the overall variability of these angles in the Ramachandran space.

As shown in Tables 2 and 3 and Figures S2-S3, the differences are very limited. The *p*-test analysis indicates that the mean values are not significantly different for the angles C^*α*^CO and C^−1^NC^*α*^. On the other hand, the local structural context has a significant impact on the angles NC^*α*^C and OCN^+1^. The influence of the local structure on the angles C^*α*^CN^+1^, NC^*α*^C^*β*^, and C^*β*^C^*α*^C shows a nonsystematic dependence on the type of secondary structure.

To further investigate the role of the conformation versus local structure, we also compared the values of these parameters for residues adopting the same structural motif (*β*-sheet) but in boxes characterized by significantly different (*ϕ*, *ψ*) angles. As shown in Table 4, differences are remarkable and statistically significant in all cases. A collective analysis of the data reported in Tables 2–4 corroborates the notion that the contribution of (*ϕ*, *ψ*) angles overcomes the impact of the local structural motif. A significant contribution of secondary structure is limited to angles which involve atoms that are usually engaged in H-bonding interactions and, therefore, more susceptible to the structural context.

### 3.2. Backbone Variability: The Impact of Residues Type

**Figure 3:**
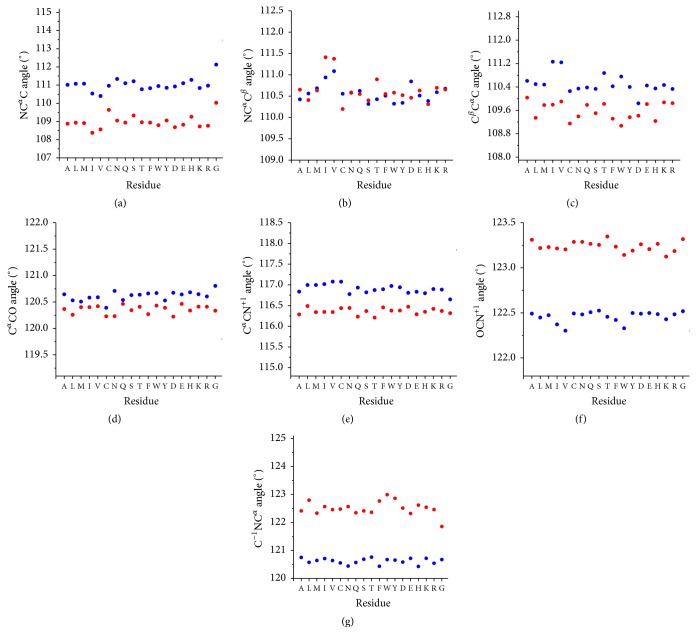
Average values of bond angles of protein residues in *α*-helix in the 3° × 3° box centered at (*φ*, *ψ*) = (−63°, −43°) (in blue) and in *β*-sheet in the 15° × 15° box centered at (*φ*, *ψ*) = (−120°, 130°) (in red) in the Ramachandran plot. Pro is not reported in the diagram since the observations for this residue are very limited.

The role of specific properties of residue side chains on the variability has been initially demonstrated by Touw and Vriend [28] and later confirmed by us [29] for the prototypical NC^*α*^C angle. We here extended these analyses to the other valence bond angles of protein backbone. In this framework, to achieve statistically significant results, we considered the highly populated (*φ*, *ψ*) boxes for the helical and the extended regions described in the previous section. It is worth mentioning that this choice ensured the occurrence of at least 100 residues of each type in the two selected boxes. The inspection of Tables 5-6 and Figures 3(a)–3(g) clearly indicates that the valence bond angles characterized by a central atom endowed with sp^2^ hybridization display a very limited dependence on the residue type. For proline, a specific value is observed for the angle C^−1^NC^*α*^ in the helical box due to the cyclic nature of this residue (Table 5). A significantly lower value is also displayed by the same angle of Gly residues in the extended context (Table 6). A more significant impact of the residue type is occasionally observed for the valence bond angles centered at the C^*α*^ atom that is spatially close to the side chain. One clear trend is observed for the angle NC^*α*^C^*β*^ which is systematically higher for the *β*-branched residues Val and Ile independently of the structural contexts (Tables 5 and 6). These latter residues also exhibit high values of the C^*β*^C^*α*^C angle, although the effect is evident only in the helical state. Since for these residues a decrease of the related NC^*α*^C angle is observed due to steric effects of the branched side chain (Tables 5-6 and [28, 29]), the enlargement of the NC^*α*^C^*β*^ and C^*β*^C^*α*^C may be a consequence of the NC^*α*^C variability. It is worth mentioning that, even in the most sterically allowed and populated rotamer of *β*-branched residues (*trans* for Val and* gauche* for Ile), the two C^*γ*^ atoms are* gauche* to both N and C atoms of their own backbone (1–4 interactions).

**Figure 4:**
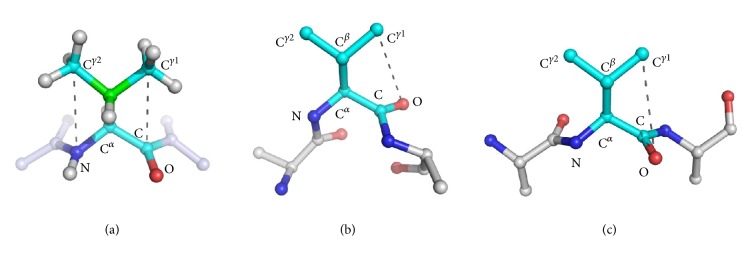
Local steric hindrance in *β*-branched residues. Ball-and-stick representations of the most populated rotamer of Val (used here as a representative model of a *β*-branched residue) are reported. (a) The backbone-independent repulsive interactions between the two heavy C^*γ*^ atoms and both backbone N and C atoms are shown as dashed gray lines; they can explain the widening of the NC^*α*^C^*β*^ and C^*β*^C^*α*^C angles (the C^*β*^ atom is shown in green). (b) The backbone-dependent contact between C^*γ*1^ atom and the O backbone atom is shown (dashed gray lines) in the *α*-helical conformation (*φ*, *ψ*) = (−63°, −43°). (c) In *β*-sheet conformation (*φ*, *ψ*) = (−120°, 130°), the same atoms are on opposite sides. In both panels (b) and (c), the H atoms are omitted for clarity.

The repulsive interactions between these groups likely produced slight displacement of the side chain with respect to the main chain. This causes an enlargement of the angles involving the C^*β*^ atom (NC^*α*^C^*β*^ and C^*β*^C^*α*^C) (Figure 4(a)). In addition to these interactions which are independent of backbone conformation, there are possible interactions displayed by the C^*γ*^ atoms due to the fact that they are in a five-atom chain (1–5 interactions) with heavy atoms whose position is determined by backbone conformation [33, 34] (Figures 4(b) and 4(c)). These (*ϕ*, *ψ*)-dependent interactions produce a slight repulsion between the C^*γ*^ atom and the O atom of the same residue in the preferred* trans* rotamer (experimental population 89%) of the *α*-helical conformation. This causes a further enlargement of the C^*β*^C^*α*^C angle in the helical conformation (Tables 5-6 and Figure 4(b)). This proximity between C^*γ*^ and O atoms does not occur in the extended conformation in the most preferred (*trans*) rotameric state (Figure 4(c)).

Other significant peculiarities are observed for the angles NC^*α*^C^*β*^ and C^*β*^C^*α*^C of proline residues (Table 5), again ascribable to its cyclic nature. Our analysis also highlights that the C^*β*^C^*α*^C tends to adopt low values for Asp residues in the helical context. The limited distortions may be due to the potential interaction that charged Asp side chains may form with the local backbone.

## 4. Conclusions

Proteins frequently assume complex three-dimensional structures characterized by marginal thermodynamic stabilities. Therefore, a full understanding of the principles underlying their folding requires a profound knowledge of all the aspects involved in this process. The variability of several geometrical parameters of protein backbone has attracted much attention and it is believed to play a role in protein folding as well as in other contexts such as structure refinement and validation. Although the structural variability of several geometrical parameters of protein backbone has been well established, the role of the structural environment in determining/modulating these effects has been hitherto limited to the prototypical *τ* (NC^*α*^C) valence bond. We here extended the analysis of the peptide backbone geometry and planarity with the aim of gaining insights into the structural determinants of this variability. As expected, present statistical surveys confirm the remarkable variability of these parameters. Collectively, present findings corroborate the notion that the contribution of (*ϕ*, *ψ*) angles overcomes the impact of the local structural motif. A significant contribution of secondary structure is limited to angles which involve atoms that are usually engaged in H-bonding interactions and, therefore, more susceptible to the structural context. In this scenario, it is not surprising that the highest dependence from the structural context is exhibited by the OCN^+1^ angle.

Present data also show that the impact of the nature of the residues' side chain is marginal in most of the cases. However, we observe that, in addition to the impact of some side chains on NC^*α*^C [28, 29], the values of angles such as NC^*α*^C^*β*^ and C^*β*^C^*α*^C may depend on the nature of residue type. In particular, these angles tend to adopt larger values in the *β*-branched residues Val and Ile. This finding may be interpreted on the basis of steric effects generated by the simultaneous presence of the bulky groups that are linked to the C^*β*^ atom. It is worth mentioning that Thr, the other *β*-branched residue, has a distinct behavior. Evidently, the presence of an oxygen atom in the Thr side chain, which may establish H-bonding interactions with the local environment, has a significant impact on the geometry of this residue. Local H-bonding interactions likely cause the peculiar values observed for the C^*β*^C^*α*^C angle of Asp in helical contexts.

In conclusion, the rather tight association between conformation and geometry explains the high energetic costs associated with the swapping of geometries between different structural states. Moreover, our analysis further corroborates the necessity of considering the variability of protein backbone geometry in structure refinement, validation, and prediction.

## Supplementary Material

Table S1: Statistical parameters derived from the linear fitting of the graphs reported in Figure 2 by considering β-sheet and α-helix structures separately. For parameters with R<0.70 the p-value has been calculated and reported in bracket. Table S2: Engh and Huber parameters for different backbone dihedral angles. The number reported in the second raw is the standard deviation. Figure S1: Ramachandran plots highlighting the experimental dependence of the bond angles NC^α^C (A), NC^α^C^β^ (B), C^β^C^α^C (C), C^α^CO (D), C^α^CN^+1^ (E), OCN^+1^(F), C^−1^NC^α^ (G) and dihedral angles Δω (H), θC (I) on backbone conformation (φ, ψ) for the eighteen non-Gly/non-Pro residues. The mean values are calculated in 5°x5° and 10°x10° (φ, ψ)-boxes for bond and dihedral angles, respectively. Only boxes containing at least 50 residues were considered. Figure S2: Distributions of bond angles values of non-Gly/non-Pro residues in α-helix (blue) or coil (grey) in the 3°x3°-box centered at (ϕ,ψ) =(-63°,-43°): NC^α^C (A), NC^α^C^β^ (B), C^β^C^α^C (C), C^α^CO (D), C^α^CN^+1^ (E), OCN^+1^(F), C^−1^NC^α^ (G). Figure S3: Distributions of bond angles values of non-Gly/non-Pro residues in β-sheet (red) or coil (grey) in the 15°x15°-box centered at (ϕ,ψ) =(-120°,130°): NC^α^C (A), NC^α^C^β^ (B), C^β^C^α^C (C), C^α^CO (D), C^α^CN^+1^ (E), OCN^+1^(F), C^−1^NC^α^ (G).

## Figures and Tables

**Table 1:** Statistical parameters of the linear fitting of the graphs reported in Figure 2. The significance in terms of *p* value is less than 10^−50^ for all parameters.

Parameter	Correlation coefficient *R*	Regression line
NC^*α*^C	0.96	*y* = 0.85*x* + 16.9
NC^*α*^C^*β*^	0.71	*y* = 0.68*x* + 35.3
C^*β*^C^*α*^C	0.88	*y* = 0.78*x* + 24.1
C^*α*^CO	0.93	*y* = 0.77*x* + 27.1
C^*α*^CN^+1^	0.93	*y* = 0.64*x* + 41.8
OCN^+1^	0.72	*y* = 0.41*x* + 72.2
C^−1^NC^*α*^	0.83	*y* = 0.72*x* + 34.5
Δ*ω*	0.89	*y* = 0.75*x* + 0.68
*θ*_C_	0.89	*y* = 0.85*x* + 0.04

**Table 2:** Average values of bond angles with standard deviations and occurrences (in bracket) of non-Gly/non-Pro residues in *α*-helix or coil in the 3°  × 3° box centered at (*φ*, *ψ*) = (−63°, −43°). The differences (coil minus *α*-helix) between the average values of the distributions and the statistical significance of these differences (*p* value) are reported.

Bond angle	〈angle〉 ± *σ*		Difference	*p* value
	*α*-Helix (11480)	Coil (181)		
NC^*α*^C	110.9 ± 1.2	111.6 ± 1.6	0.7	2.9*∗*10^−14^
C^*α*^CO	120.6 ± 0.9	120.4 ± 0.9	−0.2	0.0051
C^*α*^CN^+1^	116.9 ± 0.9	116.7 ± 1.1	−0.2	0.019
OCN^+1^	122.5 ± 0.9	122.8 ± 0.9	0.3	9.3*∗*10^−7^
C^−1^NC^*α*^	120.6 ± 1.1	120.7 ± 1.2	0.1	0.87
NC^*α*^C^*β*^	110.6 ± 1.1	110.6 ± 1.1	0.0	0.81
C^*β*^C^*α*^C	110.6 ± 1.3	110.2 ± 1.4	−0.4	8.1*∗*10^−4^

**Table 3:** Average values of bond angles with standard deviations and occurrences (in bracket) of non-Gly/non-Pro residues in *β*-structure or coil in the 15°  × 15° box centered at (*φ*, *ψ*) = (−120°, 130°). The differences (coil minus *β*-sheet) between the average values of the distributions and the statistical significance of these differences (*p* value) are reported.

Bond angle	〈angle〉 ± *σ*		Difference	*p* value
	*β*-Sheet (14076)	Coil (1968)		
NC^*α*^C	108.8 ± 1.9	109.0 ± 2.2	0.2	2.8*∗*10^−7^
C^*α*^CO	120.4 ± 0.9	120.4 ± 0.9	0.0	0.58
C^*α*^CN^+1^	116.4 ± 1.1	116.6 ± 1.1	0.2	8.4*∗*10^−16^
OCN^+1^	123.2 ± 0.9	123.0 ± 1.0	−0.2	1.3*∗*10^−22^
C^−1^NC^*α*^	122.5 ± 1.3	122.6 ± 1.4	0.1	0.15
NC^*α*^C^*β*^	110.9 ± 1.4	110.7 ± 1.4	−0.2	3.1*∗*10^−13^
C^*β*^C^*α*^C	109.7 ± 1.5	109.7 ± 1.6	0.0	0.27

**Table 4:** Average values of bond angles with standard deviations and occurrences (in bracket) of non-Gly/non-Pro residues in *β*-structure in the 15°  × 15° boxes centered at (*φ*, *ψ*) = (−120°, 130°) and (*φ*, *ψ*) = (−60°, 150°). The differences between the average values of the distributions and the statistical significance of these differences (*p* value) are reported.

Bond angle	〈angle〉 ± *σ*		Difference	*p* value
	(*φ*, *ψ*) = (−120°, 130°) (14076)	(*φ*, *ψ*) = (−60°, 150°) (680)		
NC^*α*^C	108.8 ± 1.9	110.3 ± 1.8	1.5	8.6*∗*10^−94^
C^*α*^CO	120.4 ± 0.9	121.1 ± 0.9	0.7	2.0*∗*10^−86^
C^*α*^CN^+1^	116.4 ± 1.1	115.9 ± 1.1	−0.5	2.0*∗*10^−23^
OCN^+1^	123.2 ± 0.9	122.9 ± 1.0	−0.3	3.8*∗*10^−15^
C^−1^NC^*α*^	122.5 ± 1.3	120.6 ± 1.4	−1.9	3.7*∗*10^−306^
NC^*α*^C^*β*^	110.9 ± 1.4	110.4 ± 1.4	−0.5	3.1*∗*10^−26^
C^*β*^C^*α*^C	109.7 ± 1.5	109.5 ± 1.7	−0.2	0.0025

**Table 5:** Average values (°) of the bond angles NC^*α*^C, NC^*α*^C^*β*^, C^*β*^C^*α*^C, C^*α*^CO, C^*α*^CN^+1^, OCN^+1^, and C^−1^NC^*α*^ for each amino acid residue type in *α*-helix in the 3° × 3° box centered at (*ϕ*, *ψ*) = (−63°, −43°).

Residue	Occurrence	NC^*α*^C	NC^*α*^C^*β*^	C^*β*^C^*α*^C	C^*α*^CO	C^*α*^CN^+1^	OCN^+1^	C^−1^NC^*α*^
Ala	2036	111.0	110.4	110.6	120.6	116.8	122.5	120.8
Leu	1434	111.1	110.6	110.5	120.5	117.0	122.4	120.6
Met	272	111.1	110.7	110.5	120.5	117.0	122.5	120.6
Ile	830	110.5	110.9	111.3	120.6	117.0	122.4	120.7
Val	851	110.4	111.1	111.2	120.6	117.1	122.3	120.6
Cys	122	111.0	110.6	110.3	120.4	117.0	122.5	120.6
Asn	294	111.3	110.6	110.3	120.7	116.8	122.5	120.4
Gln	650	111.1	110.6	110.4	120.5	116.9	122.5	120.6
Ser	429	111.2	110.3	110.3	120.6	116.8	122.5	120.7
Thr	491	110.8	110.4	110.9	120.6	116.9	122.5	120.8
Phe	350	110.8	110.5	110.4	120.7	116.9	122.4	120.4
Trp	183	111.0	110.3	110.8	120.7	117.0	122.3	120.7
Tyr	299	110.9	110.3	110.4	120.5	116.9	122.5	120.7
Asp	559	110.9	110.8	109.8	120.7	116.8	122.5	120.6
Glu	1002	111.1	110.5	110.4	120.6	116.8	122.5	120.7
His	208	111.3	110.4	110.3	120.7	116.8	122.5	120.4
Lys	798	110.8	110.6	110.5	120.6	116.9	122.4	120.7
Arg	746	111.0	110.7	110.3	120.6	116.9	122.5	120.5
Gly	459	112.1	—	—	120.8	116.6	122.5	120.7
Pro^*∗*^	28	113.5	103.3	111.7	119.8	117.8	122.3	117.7

^*∗*^The number of observations for Pro residues is very limited.

**Table 6:** Average values (°) of the bond angles NC^*α*^C, NC^*α*^C^*β*^, C^*β*^C^*α*^C, C^*α*^CO, C^*α*^CN^+1^, OCN^+1^, and C^−1^NC^*α*^ for each amino acid residue type in *β*-sheet in the 15° × 15° box centered at (*ϕ*, *ψ*) = (−120°, 130°). No Pro residues have been found in Data 1.6 for this box.

Residue	Occurrence	NC^*α*^C	NC^*α*^C^*β*^	C^*β*^C^*α*^C	C^*α*^CO	C^*α*^CN^+1^	OCN^+1^	C^−1^NC^*α*^
Ala	559	108.9	110.7	110.0	120.4	116.3	123.3	122.4
Leu	1717	108.9	110.4	109.3	120.3	116.5	123.2	122.8
Met	252	109.0	110.6	109.9	120.4	116.3	123.3	122.4
Ile	2816	108.4	111.4	109.8	120.4	116.3	123.2	122.6
Val	3793	108.6	111.4	109.9	120.4	116.3	123.2	122.5
Cys	182	109.7	110.2	109.3	120.2	116.5	123.3	122.5
Asn	170	109.0	110.5	109.4	120.2	116.4	123.3	122.6
Gln	325	109.0	110.6	109.7	120.5	116.2	123.3	122.3
Ser	382	109.3	110.5	109.6	120.4	116.4	123.3	122.4
Thr	1369	109.0	110.9	109.8	120.4	116.2	123.3	122.4
Phe	670	109.0	110.5	109.3	120.3	116.4	123.2	122.8
Trp	202	108.7	110.6	109.1	120.4	116.4	123.2	123.0
Tyr	624	109.1	110.5	109.4	120.4	116.4	123.2	122.9
Asp	131	108.7	110.5	109.4	120.2	116.5	123.3	122.5
Glu	575	108.8	110.7	109.8	120.5	116.3	123.2	122.4
His	245	109.2	110.3	109.2	120.3	116.4	123.3	122.7
Lys	541	108.8	110.7	109.9	120.4	116.4	123.1	122.5
Arg	580	108.8	110.6	109.8	120.4	116.4	123.2	122.5
Gly	140	110.0	—	—	120.3	116.3	123.3	121.9

## Abbreviations

PDB: Protein Data Bank
H: *α*-Helix
G: 3(10)-Helix
E: *β*-Sheet
*τ*: NC^*α*^C bond angle.
