# Supplementary material for: Dissection of Factors Affecting the Variability of the Peptide Bond Geometry and Planarity
Source: Biomed Res Int. 2017 Oct 15;2017:2617629. doi: 10.1155/2017/2617629 (PMC5661080; doi:10.1155/2017/2617629)
Supplement: Supplementary file 1 — Table S1: Statistical parameters derived from the linear fitting of the graphs reported in Figure 2 by considering β-sheet and α-helix structures separately. For parameters with R<0.70 the p-value has been calculated and reported in bracket. Table S2: Engh and Huber parameters for different backbone dihedral angles. The number reported in the second raw is the standard deviation. Figure S1: Ramachandran plots highlighting the experimental dependence of the bond angles NCαC (A), NCαCβ (B), CβCαC (C), CαCO (D), CαCN+1 (E), OCN+1(F), C−1NCα (G) and dihedral angles Δω (H), θC (I) on backbone conformation (φ, ψ) for the eighteen non-Gly/non-Pro residues. The mean values are calculated in 5°x5° and 10°x10° (φ, ψ)-boxes for bond and dihedral angles, respectively. Only boxes containing at least 50 residues were considered. Figure S2: Distributions of bond angles values of non-Gly/non-Pro residues in α-helix (blue) or coil (grey) in the 3°x3°-box centered at (ϕ,ψ) =(-63°,-43°): NCαC (A), NCαCβ (B), CβCαC (C), CαCO (D), CαCN+1 (E), OCN+1(F), C−1NCα (G). Figure S3: Distributions of bond angles values of non-Gly/non-Pro residues in β-sheet (red) or coil (grey) in the 15°x15°-box centered at (ϕ,ψ) =(-120°,130°): NCαC (A), NCαCβ (B), CβCαC (C), CαCO (D), CαCN+1 (E), OCN+1(F), C−1NCα (G). [file 2617629.f1.pdf]

Supplementary Material

Table S1. Statistical parameters derived from the linear fitting of the graphs reported in Figure 2 by considering β-sheet and α-helix structures separately. For parameters with R<0.70 the p-value has been calculated and reported in bracket.

**Parameter**	*β-sheet*		*α-helix*	
	**Correlation coefficient R**	**Regression line**	**Correlation coefficient R**	**Regression line**
$NC^{\alpha}C$	0.88	y=0.81x+21.1	0.89	y=0.49x+57.7
$NC^{\alpha}C^{\beta}$	0.76	y=0.78x+24.7	0.25 (0.0055)	y=0.25x+83.2
$C^{\beta}C^{\alpha}C$	0.93	y=0.84x+17.8	0.80	y=0.59x+44.7
$C^{\alpha}CO$	0.87	y=0.80x+24.3	0.89	y=0.66x+41.0
$C^{\alpha}CN^{+1}$	0.84	y=0.69x+36.2	0.82	y=0.82x+21.1
OCN^{+1}	0.72	y=0.63x+45.9	0.10 (0.25)	y=0.076x+113.5
$C^{-1}NC^{\alpha}$	0.78	y=0.71x+35.5	0.76	y=0.75x+30.9
$\Delta\omega$	0.92	y=0.87x+0.92	0.71	y=0.41x+1.2
θ_C	0.94	y=0.85x-0.15	0.77	y=0.62x+0.68

Table S2. Engh and Huber parameters for different backbone dihedral angles. The number reported in the second raw is the standard deviation.

Angle<br>Residue	$NC^{\alpha}C$	$C^{\alpha}CO$	$C^{\alpha}CN^{+1}$	OCN^{+1}	$C^{-1}NC^{\alpha}$	$NC^{\alpha}C^{\beta}$	$C^{\beta}C^{\alpha}C$
Pro	111.8<br>2.5	-	116.9<br>1.5	122.0<br>1.4	122.6<br>5.0	103.0<br>1.1	-
Non-Pro	-	-	-	123.0<br>1.6	-	-	-
Gly	112.5<br>2.9	120.8<br>2.1	116.4<br>2.1	-	120.6<br>1.7	-	-
Non-Gly	-	120.8<br>1.7	-	-	-	-	-
Ala	-	-	-	-	-	110.4<br>1.5	110.5<br>1.5
Ile, Thr, Val	-	-	-	-	-	111.5<br>1.7	109.1<br>2.2
Non-Gly/non-Pro	111.2<br>2.8	-	116.2<br>2.0	-	121.7<br>1.8	-	-
The rest	-	-	-		-	110.5<br>1.7	110.1<br>1.9

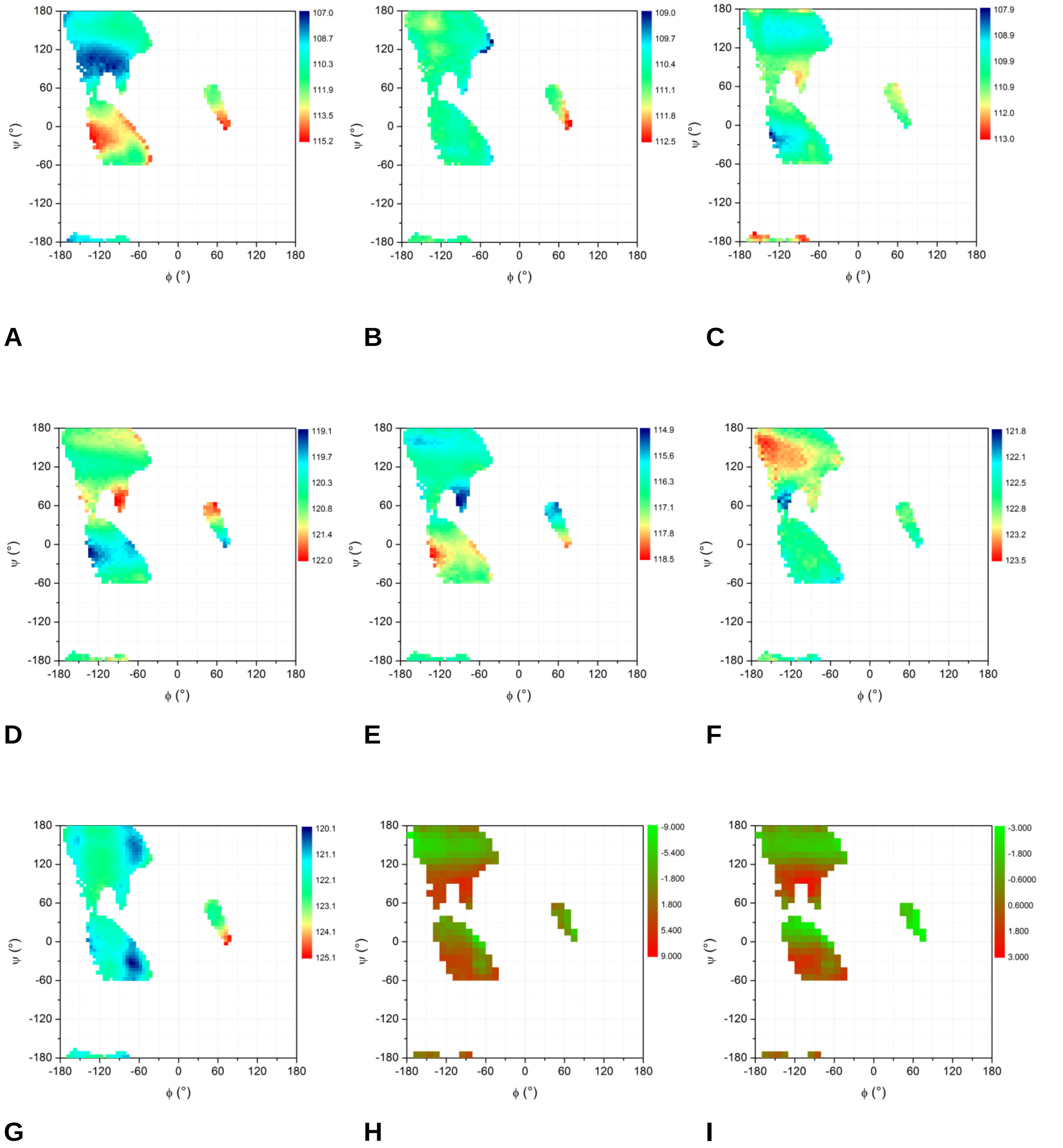

Figure S1. Ramachandran plots highlighting the experimental dependence of the bond angles $NC^{\alpha}C$ (A), $NC^{\alpha}C^{\beta}$ (B), $C^{\beta}C^{\alpha}C$ (C), $C^{\alpha}CO$ (D), $C^{\alpha}CN^{+1}$ (E), OCN^{+1}(F), $C^{-1}NC^{\alpha}$ (G) and dihedral angles $\Delta\omega$ (H), θ_C (I) on backbone conformation (φ, ψ) for the eighteen non-Gly/non-Pro residues. The mean values are calculated in 5°x5° and 10°x10° (φ, ψ)-boxes for bond and dihedral angles, respectively. Only boxes containing at least 50 residues were considered.

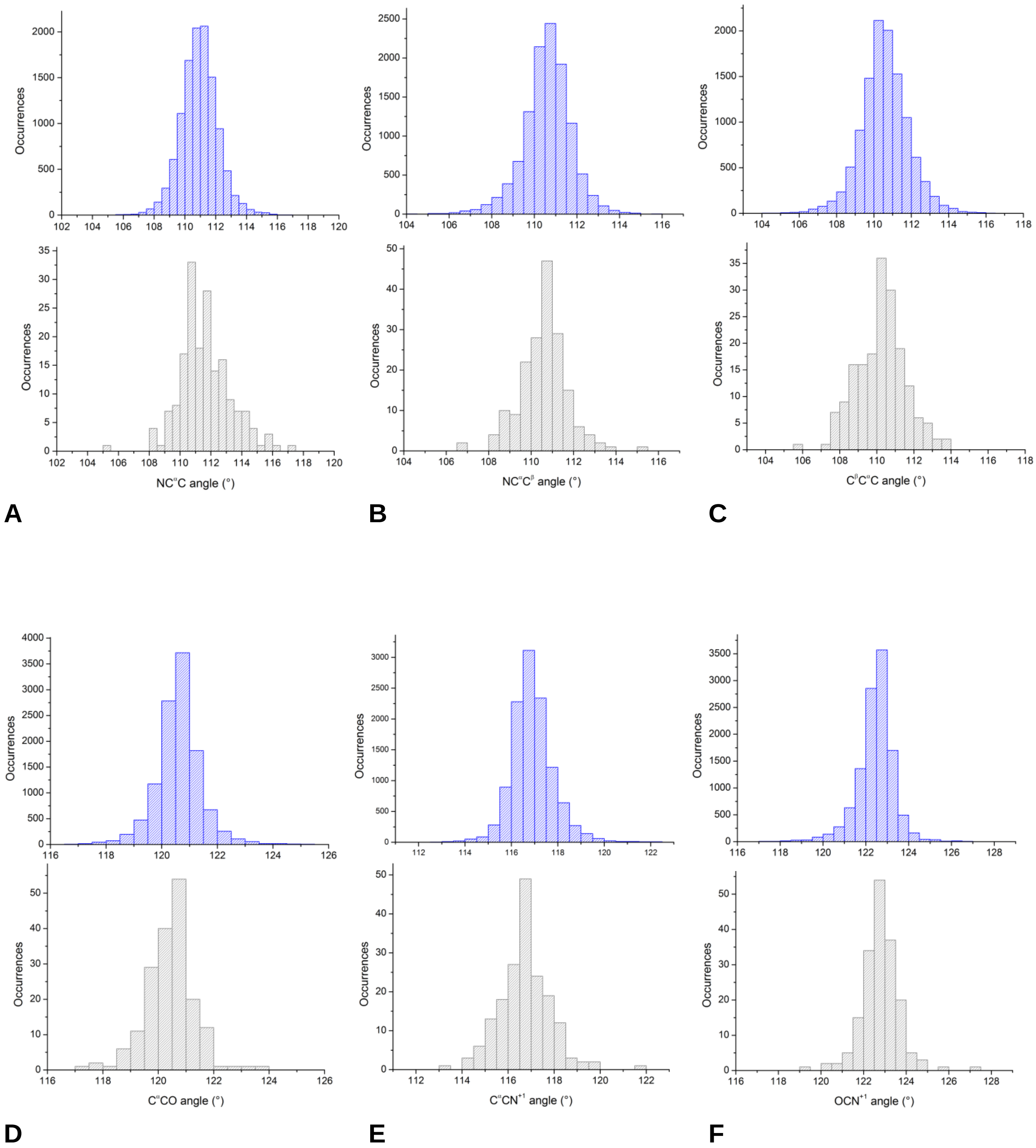

Occurrences
NCαC angle (°)
NCαCβ angle (°)
CβCαC angle (°)
CαCO angle (°)
CαCN+1 angle (°)
OCN+1 angle (°)
A
B
C
D
E
F

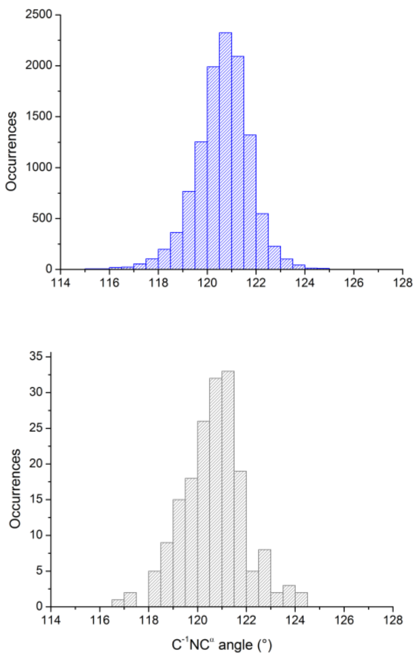

G

Figure S2. Distributions of bond angles values of non-Gly/non-Pro residues in α-helix (blue) or coil (grey) in the 3°x3°-box centered at (φ,ψ)=(-63°,-43°): $NC^{\alpha}C$ (A), $NC^{\alpha}C^{\beta}$ (B), $C^{\beta}C^{\alpha}C$ (C), $C^{\alpha}CO$ (D), $C^{\alpha}CN^{+1}$ (E), OCN^{+1}(F), $C^{-1}NC^{\alpha}$ (G).

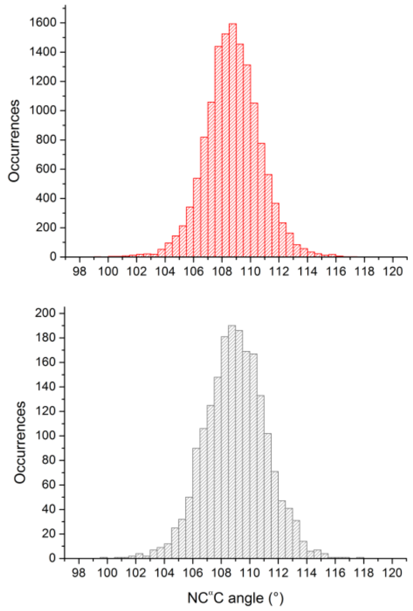
Occurrences
Occurrences
NCαC angle (°)

A

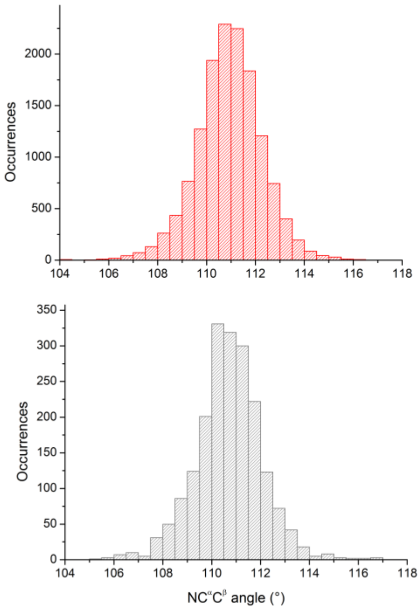
Occurrences
Occurrences
NCαCβ angle (°)

B

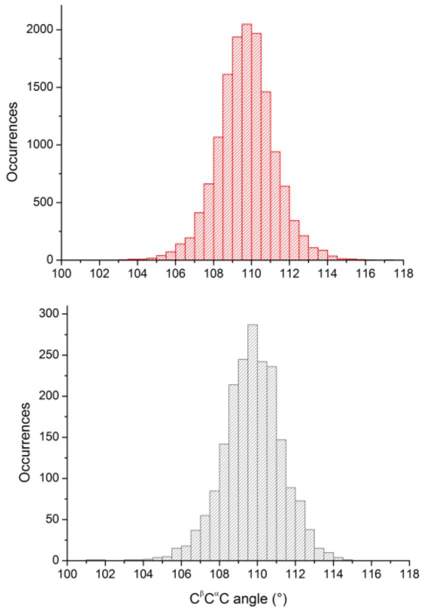
Occurrences
Occurrences
CβCαC angle (°)

C

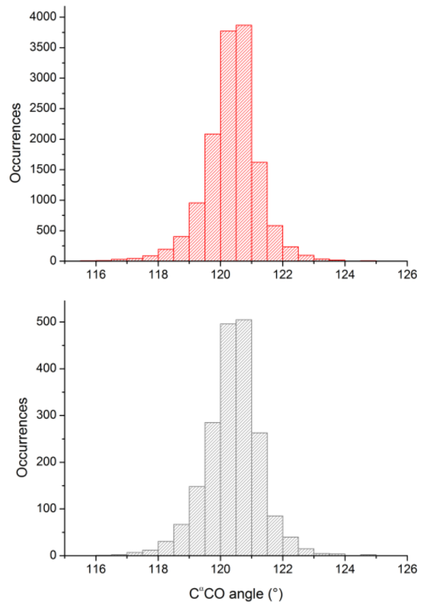
Occurrences
Occurrences
CαCO angle (°)

D

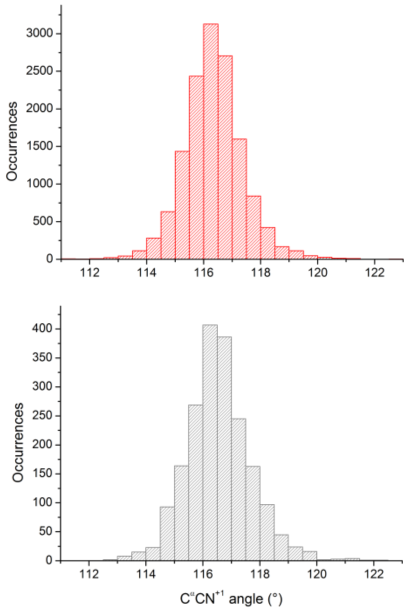
Occurrences
Occurrences
CαCN+1 angle (°)

E

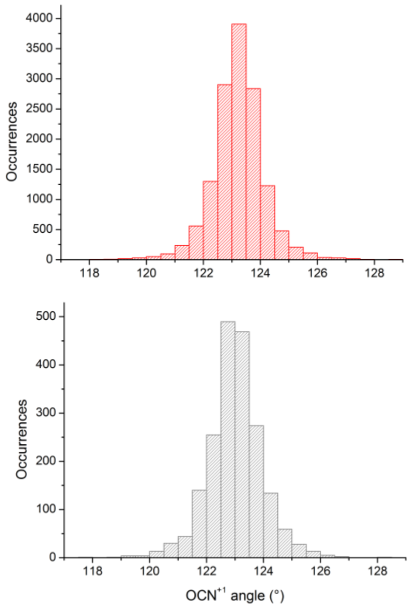
Occurrences
Occurrences
OCN+1 angle (°)

F

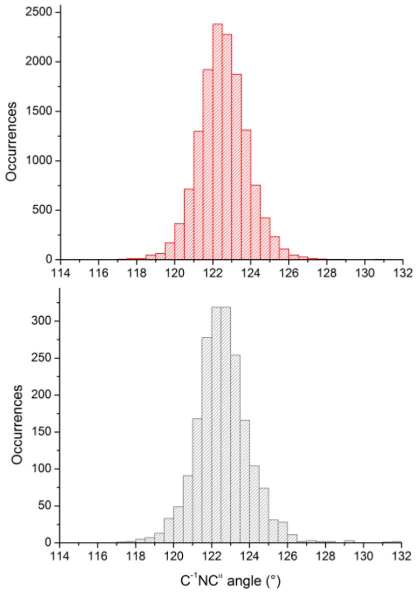

G

Figure S3. Distributions of bond angles values of non-Gly/non-Pro residues in β-sheet (red) or coil (grey) in the 15°x15°-box centered at (φ,ψ)=(-120°,130°): $NC^{\alpha}C$ (A), $NC^{\alpha}C^{\beta}$ (B), $C^{\beta}C^{\alpha}C$ (C), $C^{\alpha}CO$ (D), $C^{\alpha}CN^{+1}$ (E), OCN^{+1}(F), $C^{-1}NC^{\alpha}$ (G).
